# Supplementary material for: Pre-saccadic remapping relies on dynamics of spatial attention
Source: eLife. 2018 Dec 31;7:e37598. doi: 10.7554/eLife.37598 (PMC6328271; doi:10.7554/eLife.37598)
Supplement: Transparent reporting form [file elife-37598-transrepform.pdf]

eLife's transparent reporting form

We encourage authors to provide detailed information *within their submission* to facilitate the interpretation and replication of experiments. Authors can upload supporting documentation to indicate the use of appropriate reporting guidelines for health-related research (see EQUATOR Network), life science research (see the BioSharing Information Resource), or the ARRIVE guidelines for reporting work involving animal research. Where applicable, authors should refer to any relevant reporting standards documents in this form.

If you have any questions, please consult our Journal Policies and/or contact us: editorial@elifesciences.org.

Sample-size estimation

- You should state whether an appropriate sample size was computed when the study was being designed
- You should state the statistical method of sample size computation and any required assumptions
- If no explicit power analysis was used, you should describe how you decided what sample (replicate) size (number) to use

Please outline where this information can be found within the submission (e.g., sections or figure legends), or explain why this information doesn't apply to your submission:

No explicit power analysis was used, instead our sample size was based on different recent studies targeting similar questions with similar sample size (e.g. Yao et al, 2016; Rolfs et al. 2011).

Replicates

- You should report how often each experiment was performed
- You should include a definition of biological versus technical replication
- The data obtained should be provided and sufficient information should be provided to indicate the number of independent biological and/or technical replicates
- If you encountered any outliers, you should describe how these were handled
- Criteria for exclusion/inclusion of data should be clearly stated
- High-throughput sequence data should be uploaded before submission, with a private link for reviewers provided (these are available from both GEO and ArrayExpress)

Please outline where this information can be found within the submission (e.g., sections or figure legends), or explain why this information doesn't apply to your submission:

Each experiment (peripheral and foveal remapping tasks) was performed once. Each participant, were run in 3 to 4 repeated identical blocks per conditions (11-12 in total in the peripheral remapping task and 4 in the foveal remapping task) in a total of about 6h per participants. We therefore believe that explicit definitions of biological and technical replications do not apply to our submission. We moreover didn't exclude any participant or block from our analyses.

eLife Sciences Publications, Ltd is a limited liability non-profit non-stock corporation incorporated in the State of Delaware, USA, with company number 5030732, and is registered in the UK with company number FC030576 and branch number BR015634 at the address 1st Floor, 24 Hills Road, Cambridge CB2 1JP | August 2014

Statistical reporting

- Statistical analysis methods should be described and justified
- Raw data should be presented in figures whenever informative to do so (typically when N per group is less than 10)
- For each experiment, you should identify the statistical tests used, exact values of N, definitions of center, methods of multiple test correction, and dispersion and precision measures (e.g., mean, median, SD, SEM, confidence intervals; and, for the major substantive results, a measure of effect size (e.g., Pearson's r, Cohen's d)
- Report exact p-values wherever possible alongside the summary statistics and 95% confidence intervals. These should be reported for all key questions and not only when the p-value is less than 0.05.

Please outline where this information can be found within the submission (e.g., sections or figure legends), or explain why this information doesn't apply to your submission:

The description and justification of statistical tests used for each analysis can be found under the section "Behavioral Data analysis", lines 448-484.
For all experiments and comparisons, we report the mean and SEM normalized over the 32 tested position making the effect size directly visible, and thus a report of it redundant. We provide the effect size (partial eta-squared) of the reported ANOVA.
We report P-values were reported precisely whenever possible.

(For large datasets, or papers with a very large number of statistical tests, you may upload a single table file with tests, Ns, etc., with reference to sections in the manuscript.)

Group allocation

- Indicate how samples were allocated into experimental groups (in the case of clinical studies, please specify allocation to treatment method); if randomization was used, please also state if restricted randomization was applied
- Indicate if masking was used during group allocation, data collection and/or data analysis

Please outline where this information can be found within the submission (e.g., sections or figure legends), or explain why this information doesn't apply to your submission:

Our experiments comprise within-subject designs. This means that all subjects performed all conditions and thus no allocation into experimental groups was required.

Additional data files ("source data")

- We encourage you to upload relevant additional data files, such as numerical data that are represented as a graph in a figure, or as a summary table
- Where provided, these should be in the most useful format, and they can be uploaded as "Source data" files linked to a main figure or table
- Include model definition files including the full list of parameters used
- Include code used for data analysis (e.g., R, MatLab)
- Avoid stating that data files are "available upon request"

Please indicate the figures or tables for which source data files have been provided:

The anonymized source data can be found at: https://osf.io/3tru6/
The data analysis code can be send on request, but is not made available online as it contain the initials of the participants, not matching with the anonymized data source that we created afterward.

References

Rolfs, M., Jonikaitis, D., Deubel, H., & Cavanagh, P. (2011). Predictive remapping of attention across eye movements. *Nature Neuroscience*, *14*(2), 252–256. http://doi.org/10.1038/nn.2711

Yao, T., Ketkar, M., Treue, S., & Krishna, B. S. (2016). Visual attention is available at a task-relevant location rapidly after a saccade. *eLife*, *5*, e18009.
